# Reevaluating the Role of Corticosteroids in Septic Shock: An Updated Meta-Analysis of Randomized Controlled Trials

**DOI:** 10.1155/2019/3175047

**Published:** 2019-06-10

**Authors:** X.-J. Lian, D.-Z. Huang, Y.-S. Cao, Y.-X. Wei, Z.-Z. Lian, T.-H. Qin, P.-C. He, Y.-H. Liu, S.-H. Wang

**Affiliations:** ^1^Department of Nephrology, Guangdong Provincial People's Hospital, Guangdong Academy of Medical Sciences, Guangdong Provincial Geriatrics Institute, Guangzhou, China; ^2^Department of Gerontological Critical Care Medicine, Guangdong Provincial People's Hospital, Guangdong Academy of Medical Sciences, Guangdong Provincial Geriatrics Institute, Guangzhou, China; ^3^Department of Biostatistics, School of Public Health, Southern Medical University, Guangzhou, China; ^4^Department of Obstetrics and Gynecology, Nanfang Hospital, Southern Medical University, Guangzhou, Guangdong, China; ^5^Department of Nephrology, Guangdong Provincial People's Hospital, Guangdong Academy of Medical Sciences, Guangzhou, China; ^6^Department of Cardiology, Guangdong Cardiovascular Institute, Guangdong Provincial Key Laboratory of Coronary Heart Disease Prevention, Guangdong Provincial People's Hospital, Guangdong Academy of Medical Sciences, Guangzhou, China

## Abstract

*What Is Known and Objective. *To reevaluate the benefits and risks of corticosteroid treatment in adult patients with septic shock.* Methods.* This study was performed based on PRISMA guidelines. Randomized controlled trials (RCTs) of corticosteroids versus placebo were retrieved from PubMed, MEDLINE, EMBASE, Web of Science, the Cochrane Central RCTs, and ClinicalTrials.gov from January 1980 to April 2018. We also conducted a trial sequential analysis to indicate the possibility of type I or II errors and calculate the information size. Grading of Recommendations, Assessment, Development and Evaluation approach (GRADE) was applying to assess the certainty of evidence at the primary outcome level.* Results. *Twenty-one RCTs were identified and analyzed. Patients treated with corticosteroid had a 7% reduction in relative risk in 28-day all-cause mortality compared to controls (RR 0.93, 95% CI 0.88 to 0.99). However, there were no significant differences for the intensive care unit (ICU) mortality (RR 0.97, 95% CI 0.86 to 1.09) or in-hospital mortality (RR 1.01, 95% CI 0.92 to 1.11). Corticosteroids shortened the length of ICU stay by 1.04 days (RR -1.04, 95% CI -1.72 to -0.36) and the length of hospital stay by 2.49 days (RR -2.49, 95% CI -4.96 to -0.02). Corticosteroids increased the risk of hyperglycemia (RR 1.11, 95% CI 1.06 to 1.16) but not gastroduodenal bleeding (RR 1.06, 95% CI 0.82 to 1.37) or superinfection (RR 1.04, 95% CI 0.94 to 1.15). However, some date on secondary outcomes were unavailable because they were not measured or not reported in the included studies which may cause a lack of power or selective outcome reporting. The information size was calculated at 10044 patients. Trial sequential analysis showed that the meta-analysis was conclusive and the risk of type 2 error was minimal.* What Is New and Conclusion. *Corticosteroids are likely to be effective in reducing 28-day mortality and attenuating septic shock without increasing the rate of life-threatening complications. TSA showed that the risk of type II error in this meta-analysis was minimal and the result was conclusive.

## 1. What Is Known and Objective

Septic shock is a life-threatening condition with an extremely high short-term mortality rate ranging from 45% to 50% [1], and half of survivors may suffer from cognitive decline [2]. Several interventions have been suggested to decrease this high rate of morbidity and mortality [3–5]. Corticosteroids have pleiotropic effects in septic shock, including beneficial modulation of the immune response. The use of corticosteroids at the onset of septic shock first became standard case in the late 1970s. A half-century later, however, the safety and efficacy of corticosteroids remain controversies compared to the safety and efficacy of other adjunctive therapies [6]. Four landmark studies performed in the 1980s showed no survival benefit associated with steroids treatment for septic shock [7–10]. Nevertheless, more recent studies found potential benefits of steroids, especially regarding earlier reversal of septic shock [11–13].

A recent meta-analysis [14] provided evidence that hydrocortisone infusion or bolus may be more likely than placebo to result in shock reversal. However, no clear evidence regarding the survival benefit of any single corticosteroid or combined corticosteroid treatment regimen was found. In addition, given the 2 recent published reviews on this topic and the multitude of previous reviews, no other meta-analyses have furnished explicit evidence to support or reject the use of corticosteroid. Importantly, the sample size of the previous randomized controlled trials (RCTs) has been insufficient. Recently, the ADRENAL trial [15], a large international study, found no 90-day survival benefits associated with hydrocortisone infusion, but the infusion could speed up recovery when the septic shock was not fatal. However, the other landmark study, APROCCHSS trial [16], found a lower 90-day all-cause mortality among those who received hydrocortisone plus fludrocortisone, and this finding should certainly provoke a review of clinical practice.

Given that these new large RCTs have been published, this updated meta-analysis included these above mentioned RCTs and other RCTs identified during the updated search in order to reevaluate the efficacy and safety of corticosteroid in adults with septic shock.

## 2. Methods

### 2.1. Literature Search Strategy

This study was performed according to PRISMA guidelines and showed in Figure S1 [17]. A literature search was systematically conducted in PubMed, MEDLINE, EMBASE, Web of Science, and the Cochrane Central Register of Controlled Trials (CENTRAL) in The Cochrane Library from January 1980, because we only found one study from Schumer (1976) [18] on the literature searches for the inferior boundary (1980) and this study caused moderate heterogeneity (from I^2^=2.0%,* P*=0.43 to I^2^=30%,* P*=0.05). Moreover, based on this paper, it became standard practice in the late 1970s and early 1980s to administer high-dose corticosteroids at the onset of septic shock. The last search was run in April 2018. The search strategy is showed in Table S1. In addition, ongoing and unpublished trials were also sought through ClinicalTrials.gov. We also scanned the references list of each identified article and the references list of previous meta-analyses on the topic [14, 19–23]. There were no restrictions on language. The registration number for this meta-analysis was PROSPEROCRD42018092535. We were unaware of unpublished/ongoing studies during literature searches. In addition, we also presented a clear summary of previous meta-analyses findings, which may be helpful for reference (Table S2).

### 2.2. Study Selection

All identified titles and abstracts were assessed by two independent reviewers (DZH and XJL). Only studies that were clearly irrelevant were excluded. Disagreements were settled through discussion with a third reviewer (YHL). RCTs comparing the outcome of corticosteroid treatment vs placebo in adult with septic shock were included. The following exclusion criteria were used: (1) non-RCTs, (2) children (<18 years), (3) studies in which both groups received steroids, studies lacking information on the exact treatment regimens, or studies lacking information on the septic shock outcomes, (4) duplicate data, and (5) in vitro or preclinical animal studies. Studies designed to investigate sepsis or severe sepsis but which did not have separate data on septic shock were also excluded, after attempting to obtain the separate data from the authors.

### 2.3. Outcome

The primary outcomes were 28-day all-cause mortality. The secondary outcomes were as follows: other mortality (intensive care unit [ICU] mortality and in-hospital mortality), duration of mechanical ventilation, the length of ICU and hospital stay, and the incidence of gastrointestinal bleeding, superinfection, and hyperglycemia.

### 2.4. Data Extraction and Quality Assessment

Data extraction was conducted by two independent reviewers. Relevant data from the eligible studies were extracted by one reviewer (XJL) and checked by the other reviewer (DZH). For each included study, a record of the first author, publication date, number of study sites, location, participant characteristics (number of participants, mean age, and proportion of males), treatment, comparator, and clinical outcomes was extracted. A summary of the recorded patient data is presented in Table 1.

The methodological quality and risk of bias within each individual trial were independently assessed by two reviewers (XJL and HDZ), according to The Cochrane Handbook for Systematic Reviews of Interventions [24]. The disagreements were settled by discussion between the reviewers and adjudicated by a third reviewer (LYH). We used the Grading of Recommendations Assessment, Development, and Evaluation (GRADE) approach to assess the overall quality of evidence for primary outcome measure [25], which was presented in Figure S2.

### 2.5. Statistical Analysis

We analyzed the data by using Review Manager version 5.3 (The Nordic Cochrane Centre, Copenhagen, Denmark) and Stata version 14 (Stata Corp LP, College Station, TX, USA). Relative risk (RR) with 95% confidence interval (CI) was used for the dichotomous outcomes and weighted mean difference (MD) with 95% CI was used for the continuous outcomes. The statistical variables Q and* I*^*2*^ were used to compare the heterogeneity among studies.* I*^2^ values <25%, 25–50%, and >50% were considered to represent low, moderate, and severe heterogeneity. A fixed-effects model was applied if there was minimal significant heterogeneity. Otherwise, a random-effects model was applied. In addition, funnel plots and Egger's and Begg's tests were used to assess publication bias. Moreover, several subgroup analyses were conducted to identify potential differences in treatment effects across the trials based on treatment factors (i.e., dose, duration, and whether a concomitant mineralocorticoid was used), date of publication, and sample size. Leave-one-out sensitivity analysis was also performed to evaluate the robustness of the results. All tests were two-tailed, and* P*<0.05 was considered statistically significant in the meta-analysis.

### 2.6. Trial Sequential Analysis (TSA)

We performed a TSA for one of the primary outcomes (28-day all-cause mortality) using TSA software version 0.9.5.10 Beta (Copenhagen Trial Unit, Copenhagen, Denmark). We planned to maintain an overall risk of a type 1 error of 5% and a power of 80%. The risk of type 1 error was controlled by using the O'Brien-Fleming *α*-spending function, which indicates statistical significance if a conventional Z-curve crosses the O'Brien-Fleming *α*-spending boundaries. The risk of type II error was controlled using the *β*-spending function and futility boundaries.

## 3. Results

### 3.1. Number of Included Studies

**Figure 1:**
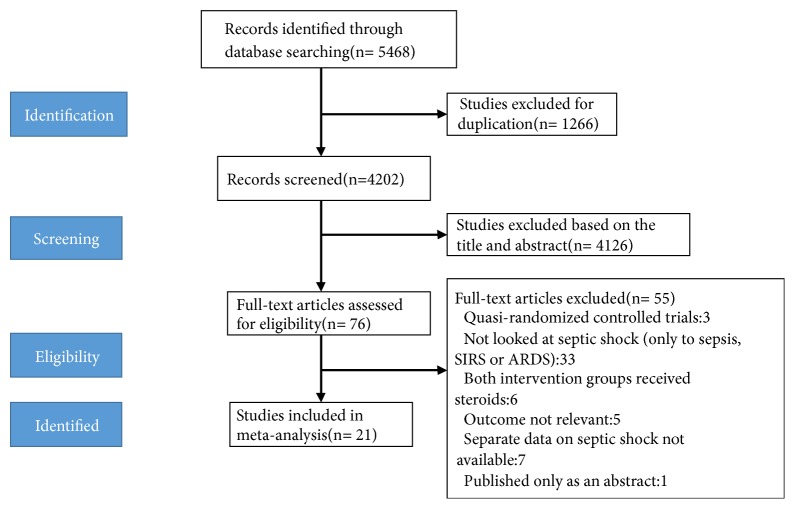
Study flow diagram.

A flowchart of the literature search is shown in Figure 1. The literature search yielded 5468 articles, of which 76 underwent a full-text review. Of these, 55 were further excluded. Consequently, 21 RCTs were finally included.

### 3.2. Study Characteristics and Interventions

A total of 9,043 patients were included. Of these, corticosteroids were given to 4,532 and 4,511 served as controls. The mean patient age ranged from 47 ± 4 to 69 ± 11 years. Ten trials [7, 9, 11, 13, 15, 16, 26–29] were multicenter, and 11 trials [10, 12, 30–38] were single center. Eighteen trials [7, 11–13, 15, 16, 26–35, 37, 38] included only patients with septic shock, while 3 trials [9, 10, 36] included patients with severe sepsis or septic shock, and separate data for the septic shock patients were available. The most common corticosteroid used was hydrocortisone (200–300 mg per day in divided doses), which was used in 14 trials. Hydrocortisone alone was used in 14 trials [12, 13, 15, 26, 27, 29–32, 34–38] while only 2 trials [11, 16] evaluated the influence of concomitant use of fludrocortisones (50 mg per day). Lastly, 18 trials [11–13, 15, 16, 26–38] investigated a prolonged course of low-dose intravenous hydrocortisone while 3 trials [7, 9, 10] investigated a short course of high-dose corticosteroids.

### 3.3. Assessment of Study Quality and Publication Bias

**Figure 2:**
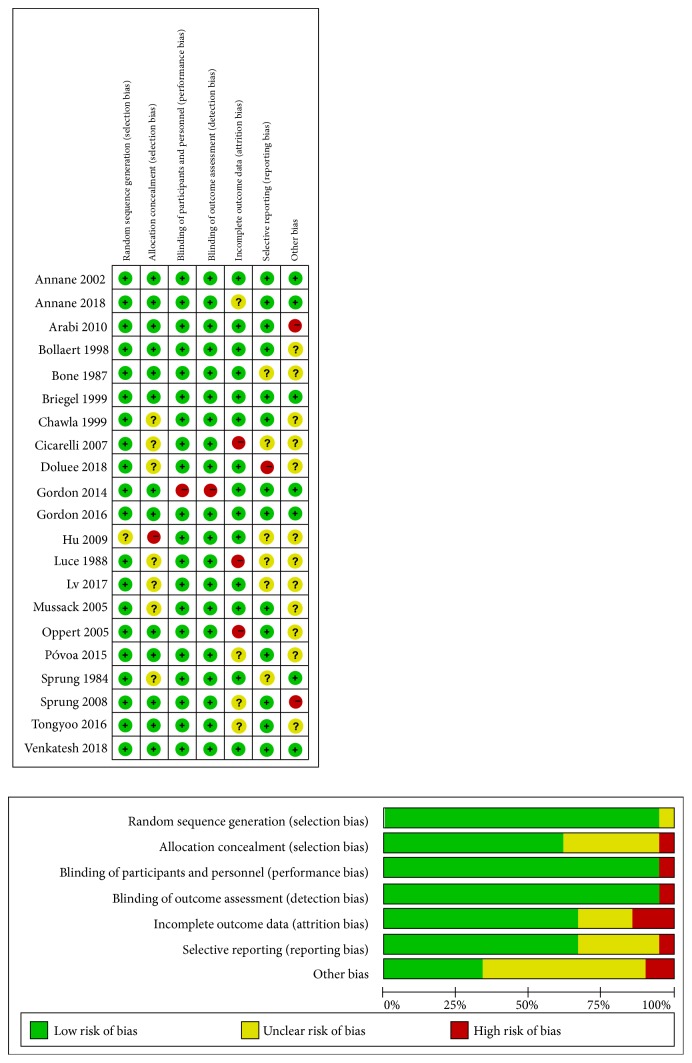
Risk of bias summary and graph in each domain for individual studies. (Green + = adequate. Red - = inadequate. Yellow? = unclear). Other biases refer to either academic or funding bias.

**Figure 3:**
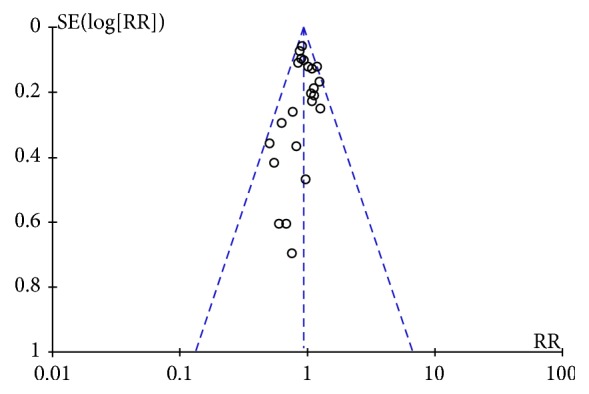
Funnel plot with 95% confidence interval (CI) to assess publication bias.

The methodological quality assessment results for each included study are outlined graphically in Figure 2. There was no apparent systematic publication bias among the included trials, based on the result of Egger's test. The* P* value was 0.891 for 28-day mortality. The funnel plot was relatively symmetrical (Figure 3).

### 3.4. All-Cause Mortality

**Figure 4:**
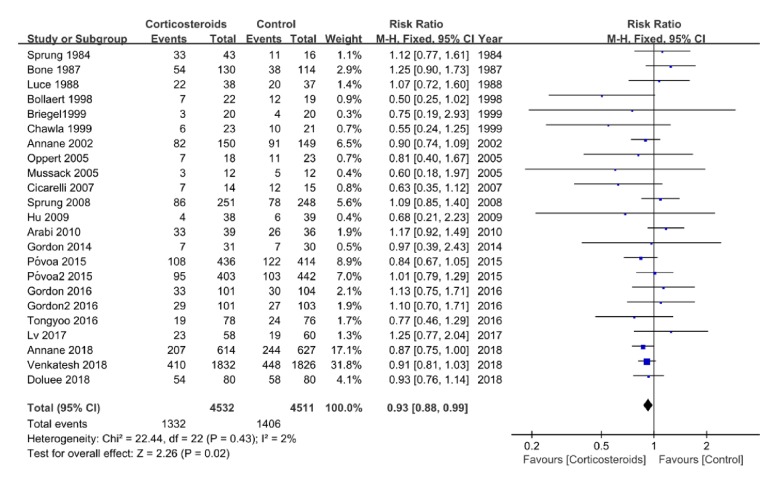
Forest plots of comparison corticosteroids versus control for 28-day all-cause mortality.

Data on 28-day all-cause mortality were available in all trials, while data on 90-day mortality were only available in 4 trials. In addition, 9 trials recorded ICU mortality and 13 trials recorded in-hospital mortality. Participants taking corticosteroids had a 7% reduction in relative risk in 28-day all-cause mortality compared to controls, according to a fixed-effects model (RR 0.93, 95% CI 0.88 to 0.99,* P*=0.02), with minimal heterogeneity (*I*^2^=2.0%,* P*=0.43) (Figure 4). However, there were no significant differences between the two groups regarding ICU mortality (RR 0.97, 95% CI 0.86 to 1.09,* P*=0.56;* I*^*2*^=0%,* P*=0.49) or in-hospital mortality (RR 1.01, 95% CI 0.92 to 1.11,* P*=0.85;* I*^*2*^=0%,* P*=0.80) (Figure S3).

### 3.5. Length of ICU or Hospital Stay

We were able to extract data on length of ICU stay from 12 trials and length of hospital stay from 7 trials. There were two studies that presented the relevant data as medians and interquartile ranges. We treated the median as similar as the mean and the width of the interquartile range as similar as approximately 1.35 standard deviations, according to the Cochrane Handbook. Compared to the control group, the corticosteroid group had a shortened length of ICU stay, by 1.04 days, in a fixed-effects model (MD -1.04, 95% CI -1.72 to -0.36,* P*=0.003), with low heterogeneity across studies (*I*^2^=25%,* P*=0.19). In addition, the corticosteroid group had tendency to have a shortened length of hospital stay, by 2.49 days, in a fixed-effects model (MD -2.49, 95% CI -4.96 to -0.02,* P*=0.05), with no heterogeneity across studies (*I*^2^=0%,* P*=0.75) (Figure S4).

### 3.6. Mechanical Ventilation

Data on the number of mechanical ventilation-free days and the median time to cessation of initial mechanical ventilation were available from 4 trials. Participants taking corticosteroids had significantly more mechanical ventilation-free days than the controls, based on a fixed-effects model (RR 1.07, 95% CI 0.07 to 2.08,* P*=0.04). Participants taking corticosteroids also had a shorter duration of initial mechanical ventilation compared to the controls, based on a fixed-effects model (MD -0.89, 95% CI -1.60 to -0.18,* P*=0.01). For both analyses, there was no heterogeneity across studies (*I*^2^=0%* P*=0.57,* P*=0.91) (Figure S5).

### 3.7. Adverse Events of Therapy

Gastroduodenal bleeding (based on data from 10 trials) was observed in 102 of 3032 (3.36%) participants in the corticosteroid group vs. 94 of 2999 (3.13%) participants in the control group (RR 1.06, 95% CI 0.82 to 1.37,* P*=0.66, fixed-effects model), with low heterogeneity across studies (*I*^2^=8%,* P*=0.37). Superinfections (based on data from 12 trials) were observed in 639 of 3176 (20.12%) participants in the corticosteroid group vs. 603 of 3128 (19.28%) participants in the control group (RR 1.04, 95% CI 0.94 to 1.15,* P*=0.41, fixed-effects model), with no heterogeneity across studies (*I*^2^=0%,* P*=0.61). Furthermore, the incidence of hyperglycemia (based on data from 8 trials) in the corticosteroid group was higher than in the control group (30.98% vs 28.33%, RR 1.11, 95% CI 1.06 to 1.16,* P<0*.001, fixed-effects model), with no heterogeneity across studies (*I*^2^=0%,* P*=0.63) (Figure S6).

### 3.8. Subgroup Analysis

The results of several subgroup analyses are shown in Table 2. In trials evaluating long courses of low-dose corticosteroids, there was a clear corticosteroid treatment effect on 28-day mortality (RR 0.93, 95% CI 0.87 to 0.98,* P*=0.01), with no heterogeneity across trials (*I*^2^=0%,* P*=0.52). In trials evaluating hydrocortisone plus fludrocortisone, there was a clear corticosteroid treatment effect on 28-day mortality (RR 0.87, 95% CI 0.78 to 0.99,* P*=0.03), with no heterogeneity across trials (*I*^2^=0%,* P*=0.79). In trials published in or after 2000, there was a beneficial corticosteroid treatment effect on 28-day mortality (RR 0.93, 95% CI 0.88 to 0.99,* P*=0.03), with no heterogeneity across trials (*I*^2^=0%,* P*=0.64). In trials with sample sizes >400, there was also a beneficial corticosteroid treatment effect on 28-day mortality (RR 0.90, 95% CI 0.83 to 0.98,* P*=0.01), with no heterogeneity across trials (*I*^2^=0%,* P=*0.67). However, subgroup analyses of trials evaluating short courses of high-dose corticosteroids, trials evaluating hydrocortisone without fludrocortisone, trials published before 2000, and trials with sample size ≤400 showed no survival benefits regarding 28-day mortality (Figure S7). Indeed, the subgroup analyses of sample size and hydrocortisone concomitant fludrocortisone therapy were not preregistered. A new large RCT [16] that accessed hydrocortisone plus fludrocortisone for adults with septic shock was published after registration. In addition, during data extraction, sample size of different studies showed huge fluctuations ranged from 24 (Mussack 2005 [32]) to 3658 (Venkatesh 2018 [15]) and different survival benefits on subgroup analysis. Therefore, we then believed it is significant to supply sample size and hydrocortisone concomitant fludrocortisone therapy into subgroup analysis even after registration.

### 3.9. Trial Sequential Analysis

**Figure 5:**
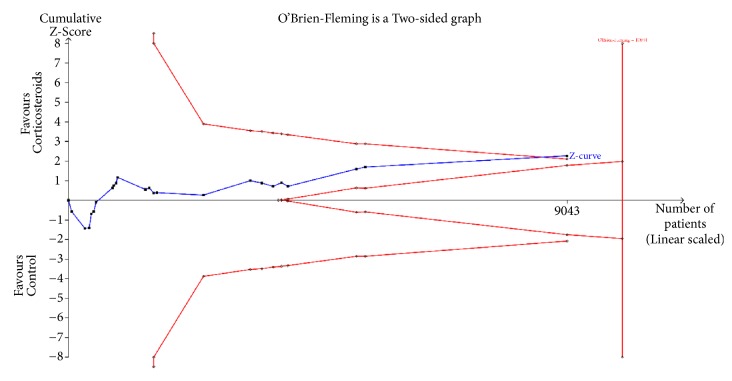
Results of trial sequential analysis for 28-day all-cause mortality. A diversity-adjusted information size of 10044 patients was calculated based on an anticipated RRR of 7% (event proportion of 40% in the control arm, *α*=0.05 [two-sided]; *β*=0.20 [power 80%]).

We estimated the information size for the analyses based on the achievement of 80% power and a 7% relative risk reduction between the corticosteroid and control groups. The incidence in the control group used in the estimation of the information size was 40%, which was estimated using a random-effects meta-analysis model. The assumed relative risk reduction of 7% in the corticosteroid group was the result of a fixed-effects model (Figure 5). TSA showed that the meta-analysis was conclusive and the risk of type II error was minimal.

## 4. Discussion

The present updated meta-analysis demonstrated the following results. First, corticosteroid treatment was associated with a 7% reduction in relative risk in 28-day all-cause mortality, and corticosteroid treatment may attenuate septic shock, as reflected in shorter hospital or ICU stays and shorter duration of mechanical ventilation. However, there is no clear significant corticosteroid effect on ICU or in-hospital mortality. Finally, corticosteroids increase the risk of developing hyperglycemia, but no significant differences in the incidence of gastrointestinal bleeding or superinfection were found.

Previous meta-analyses [14, 22, 39] have evaluated the effect of corticosteroids on mortality among patients with septic shock, but they did not find clear evidence that corticosteroids could reduce 28-day all-cause mortality. Our conclusion contrasts with the conclusion of these previous meta-analyses, suggesting beneficial effects related to the use of corticosteroids. The two major reasons for the contrasting conclusions were as follows. First, this analysis was limited to only RCTs and patients with septic shock, which may contrast with the inclusion criteria of other meta-analyses. For example, a meta-analysis by Sligl et al. [22] from 2009 included 8 studies, of which 2 (by Levy et al. [40] and Raurich et al. [41]) were retrospective cohort study and were excluded from our analysis. In addition, a network meta-analysis by Gibbison et al. [14] from 2017 included 22 studies, 14 of which were included in our analysis while 8 were excluded because they were not restricted to patients with septic shock or did not provide specific mortality rates for the septic shock subpopulation. Moreover, the newly published meta-analysis by Zhu et al. [42] included fewer researches than the present study. And one of the included studies [43] was excluded in our study because of not including the patients with septic shock. Another meta-analysis by Rygard et al. [44] only assessed the role of low-dose corticosteroids on outcomes. In addition, one study by CSG et al. [45] included the children; one study by Tandan et al. [46] did not report the treatment regimens and another study [47] was only designed to evaluate the respiratory function in pneumonia. Therefore, these studies were excluded in our study. Furthermore, in our meta-analysis, a fixed-effects model was used because minimal heterogeneity was found in the analysis of 28-day all-cause mortality (RR 0.93, 95% CI 0.88 to 0.99,* P*=0.01;* I*^2^=2.0%,* P*=0.43). In contrast, the previous meta-analyses tended to use a random-effects model due to the high level of heterogeneity across studies, and they produced conservative results. Furthermore, in our meta-analysis, a fixed-effects model was used because minimal heterogeneity was found in the analysis of 28-day all-cause mortality (RR 0.93, 95% CI 0.88 to 0.99,* P*=0.01;* I*^2^=2.0%,* P*=0.43). In contrast, the previous meta-analyses tended to use a random-effects model due to the high level of heterogeneity across studies, and they produced conservative results. Nevertheless, the random-effects model in our study still showed a tendency toward a 28-day survival benefit (RR 0.94, 95% CI 0.89 to 1.00,* P*=0.05).

Second, and most importantly, almost all the previous RCTs included in meta-analysis had small sample sizes. TSA of recent meta-analysis [48], which did not include recent two large-scale and high-quality studies [15, 16], demonstrated that there remained lacking evidence to draw a firm conclusion on the corticosteroid's effect on mortality. However, TSA in the present updated meta-analysis showed the result was conclusive.

In the modern era, there has been significant evolution in how corticosteroids are administered. In particular, lower-dose hydrocortisone has become ever more common [49]. Thus, we divided the studies according to whether they were published before or during the 21st century, as well as whether they involved a long course of low-dose or a short course of high-dose corticosteroid treatment. Subgroup analysis showed both post-21st century treatment and long courses of low-dose corticosteroids decreased 28-day all-cause mortality. However, current clinical practice guidelines on the use of hydrocortisone for septic shock still indicate that the associated evidence is weak due to the low-quality nature of evidence [50].

In addition, fludrocortisone has been previously shown to be ineffective [51]. In contrast, our subgroup analysis of hydrocortisone used concomitantly with fludrocortisone showed a survival benefit (RR 0.87, 95% CI 0.78-0.99,* P*=0.03). These findings are in accordance with the findings of the first trial that added fludrocortisone to hydrocortisone in order to provide additional mineralocorticoid potency (Ger-Inf-05) [11]. This trial showed significant survival benefit from a 28-day course of hydrocortisone plus fludrocortisone compared to placebo. Similarly, a more recent second trial (APROCCHSS), involving 1241 adults with septic shock, showed lower 90-day all-cause mortality among patients who received hydrocortisone plus fludrocortisone compared to placebo. The number of relevant studies on hydrocortisone plus fludrocortisone remains insufficient. Hence, there continues to be no conclusive evidence that this combination treatment could be used as a routine treatment in adult patients with septic shock.

In terms of the complications of corticosteroids, we obtained similar results to previous studies [19, 22, 23] in that corticosteroids were shown to increase blood glucose levels. However, corticosteroids did not increase the risk of superinfection or gastrointestinal bleeding. These results may be important for clinical practice because corticosteroids could be useful if they could attenuate septic shock while not significantly increasing the risk of adverse events. However, the trial sample sizes related to the adverse events analysis were small, so additional trials with increased sample sizes are needed to provide further evidence.

The present study had several limitations. First, because different grading systems were used to compare disease severity among the included trials, it was difficult to evaluate the between-trial differences in disease severity, which may have caused heterogeneity. Second, one included trial [31] was published only as an abstract. Third, the effects on heterogeneity of different sources of infection and different primary causes of septic shock were unclear. Fourth, the sample sizes were still insufficient and the data on some reported outcomes were not fully available. Finally, some date on secondary outcomes were missing because they were not measured or no reported in the included studies, which may cause a lack of power or selective outcome reporting. However, where possible, if missing data are encountered, we will attempt to contact the individual study authors for additional information, if not, we had to make the results with the help of core outcome set existed in the field [20]. We believed such a core outcome set could be further developed. Despite these limitations, this meta-analysis included the new large RCTs and was restricted to only adult patients with septic shock in order to reevaluate the role of corticosteroids in modern medicine.

## 5. What Is New and Conclusion

Treatment with corticosteroids can decrease the risk of 28-day mortality and attenuate septic shock without significantly increasing life-threatening complications. Furthermore, TSA showed that the risk of type II error in this meta-analysis was minimal and the result was conclusive.

## Figures and Tables

**Table 1:** Baseline characteristics of included studies and population.

Study (year)	Location	Design	No. of research centres	No. of participants	Participants	Age corticosteroids vs placebo	Male corticosteroids vs placebo	Interventions	Clinical outcomes
Sprung et al,1984	USA	Multicentre double blind randomised placebo controlled trial	2	59	Adults Vasopressor dependent shock	55(4) vs 48(4)	36% vs 10%	Dexamethasone 6mg/kg Methylprednisolone 30mg/kg Doses could be repeated	Hospital mortality, shock reversal, complications of septic shock, and adverse events

Bone et al, 1987	USA	Multicentre double blind randomised placebo controlled trial	19	382	Adults With sepsis (n=234) OR Septic shock (n=148)	53(16) vs 53.7(16)	NA	Methylprednisolone 30mg/kg Every 6 hours Duration: 24hrs	For septic shock: shock reversal, 14-d mortality, and adverse events

Luce et al, 1988	USA	Single-centre double blind randomised placebo controlled trial	1	75	Adults (n=75) Sepsis AND septic shock	50(2.5) vs 53(2.5)	68.4% vs 83.8%	Methylprednisolone 30mg/kg every 6hrs Duration: 1 day	Prevention of ARDS and hospital mortality

Bollaert et al, 1998	France	Multicentre double blind randomised placebo controlled trial	2	41	Adults Vasopressor AND ventilator dependent septic shock	66(21,83) vs 56(34,81)	68.2% vs 63.2%	Hydrocortisone 100mg Every 8hrs Duration: 5 days then tapered over 6 days	Shock reversal, 28-d, ICU and hospital mortality, improvement in haemodynamics, ICU and hospital LoS, and adverse events

Briegel et al, 1999	Germany	Single-centre double blind randomised placebo controlled trial	1	40	Adults Vasopressor AND Ventilator dependent septic shock	47(4) vs 51(5)	45% vs 60%	Hydrocortisone 100mg loading 0.18mg/kg/hr maintenance Duration: Until shock reversal, then tapered off	Shock reversal, 28-d, hospital and ICU mortality, Improvement in haemodynamics, ICU and hospital LoS, and adverse events

Chawla et al, 1999	USA	Single-centre double blind randomised placebo controlled trial	1	44	Adults Vasopressor dependent shock	NA	NA	Hydrocortisone 100mg Every 8hrs Duration: 3 days then tapered over 4 days	Shock reversal, 28-d and hospital mortality, improvement in haemodynamics, ICU LoS, and adverse events

Annane et al, 2002	France	Multicentre double blind randomised placebo controlled trial	19	300	Adults Vasopressor AND Ventilator dependent septic shock.	60(17) vs 62(15)	70% vs 64%	Hydrocortisone 50mg Every 6hrs AND Fludrocortisone 50mcg Every 24hrs Duration 7 days	28-d, ICU, hospital and 1-y mortality, shock reversal, organ system failure free days, ICU and hospital LoS, and adverse events

Oppert et al, 2005	Germany	Single-centre double blind randomised placebo controlled trial	1	40	Adults Vasopressor dependent septic shock	59 vs 47	72% vs 83%	Hydrocortisone Load: 50mg Maint: 0.18mg/kg/hr Duration: until stopping vasopressor 0.06mg/kg/hr for 1 day then reduced by 0.02mg/kg/hr every day	Shock reversal, 28-d mortality, cytokine response, and SOFA score

Mussack et al, 2005		Single-centre double blind randomised placebo controlled trial	1	24	Adults Vasopressor dependent septic shock	41(30,52) vs 54 (47,57)	58% vs 50%	Hydrocortisone Load: 100mg Maint: 0.18 mg/kg/h for Duration 6 days.	28-d mortality and shock reversal

Cicarelli et al, 2007	Brazil	Single-centre double blind randomised placebo controlled trial	1	29	Adults Vasopressor dependent septic shock	69(11) vs 61(15)	42.9% vs 46.7%	Dexamethasone 0.2mg/kg Every 36hrs Duration: 3 doses	28-d mortality Duration of vasopressor support Duration of mechanical ventilation

Sprung et al, 2008	Europe Israel	Multicentre double blind randomised placebo controlled trial	52	499	Adults Septic shock	63(14) vs 63(15)	34% vs 33%	Hydrocortisone 50mg every 6hrs – 5 days 50mg every 12hrs – 3 days 50mg every day – 3 days	28-d, ICU hospital and 1-y mortality, shock reversal, organ failure–free days, and adverse events

Hu et al, 2009	China	Single-centre double blind randomised placebo controlled trial	1	77	Adults Septic shock	56.17(33.85) vs 54.91(35.36)	61% vs 64%	Hydrocortisone 50mg every 6hrs for 7 days 50mg every 8hrs for 3 days 50mg every 12hrs for 2 days 50mg every 24hrs for 2 days	Time on noradrenaline lactate clearance and ICU mortality and ICU LoS, and shock reversal

Arabi et al, 2010	Saudi Arabia	Single-centre double blind randomised placebo controlled trial	1	75	Adults Liver cirrhosis and septic shock	60.6(12.6) vs 59.3(12.2)	44% vs 44%	Hydrocortisone 50mg Every 6hrs Duration: Until shock resolution then tapered by 10mg every 48hrs until stopped	28-d, ICU and hospital mortality, shock reversal, mechanical ventilation free days, RRT free days, ICU and hospital LoS, SOFA Score at d7, and adverse events

Gordon et al, 2014	UK	Multicentre prospective open-label randomized controlled pilot trial	4	61	Adults Septic shock treated with vasopressor	61 (54, 68) vs 60 (48, 76)	58% vs 60%	Hydrocortisone 50mg Every 6 hours for 5 days Every 13hrs for 3 days Every 24hrs for 3 days	Vasopressin requirements, 28-d, ICU and hospital mortality, organ failure free days, shock reversal, ICU and hospital LoS, and adverse events

Póvoa et al, 2015	Portugal	Multicentre double blind randomised placebo controlled trial	?	1695	Adults Septic shock treated with vasopressor	For DrotAA + steroid vs DrotAA+ steroid: 64.4 (52.5, 74.2) vs 66.2 (55.4, 76.0). For placebo +steroid vs placebo: 66.2 (54.5, 76.6) vs 63.6 (51.4, 75.2)	For DrotAA + steroid vs DrotAA:56.4% vs 59.2%. For placebo +steroid vs placebo:54.1% vs 56.1%.	DrotAA 24 *μ*g/kg/hour and steroids at baseline. Placebo and steroids at baseline	28-d and 90-d mortality, SOFA Score

Gordon et al, 2016	UK	Multicentre double blind randomised placebo controlled trial	18	409	Adults Septic shock	For vasopressin+ hydrocortisone vs vasopressin+ Placebo: 66 (57,76) vs 67 (59,77). For norepinephrine + Hydrocortisone vs Norepinephrine + Hydrocortisone+ Placebo: 63 (52,76) vs 63 (52,76).	For vasopressin+ hydrocortisone vs vasopressin+ Placebo: 58% vs 50%. or norepinephrine + Hydrocortisone vs Norepinephrine + Hydrocortisone+ Placebo: 61% vs 63%.	hydrocortisone 50 mg Every 6 hours for 5 days Every 12 hours for 3 days Once daily for 3 days	kidney failure–free days, rates and duration of renal replacement therapy, 28-d, ICU and hospital mortality, SOFA Score, and adverse events

Tongyoo et al, 2016	Bangkok	Single-centre double blind randomised placebo controlled trial	1	197	Adults With sever sepsis (n=43) OR Septic shock (n=154)	64.5(17.3) vs 64.3(16.0)	51% vs 51.5%	hydrocortisone 50 mg Every 6 h daily for 7 days.	28-d and 60-d mortality and 28-day survival without organ support

Lv et al, 2017	China	Single-centre double blind randomised placebo controlled trial	1	118	Adults Septic shock treated with norepinephrine	68.8(12.6) vs 64.8(16.7)	55.2% vs 61.7%	hydrocortisone 200* *mg/d for 6 days 100mg/d for 3 days 50mg/d for 3 days	28-d and hospital mortality, shock reversal, ICU and hospital LoS

Doluee et al, 2018	Iran	Single-centre double blind randomised placebo controlled trial	1	160	Adults refractory septic shock treated with vasopressor	67.13(10.92) vs 66.93(11.2)	58.8% vs 41.3%	Hydrocortisone 50mg iv every 6 hours for 7 days	28-d mortality and return of shock

Annane et al, 2018	France	Multicentre double blind randomised placebo controlled trial	18	1241	Septic shock treated with vasopressor	66(14) vs 66(15)	65.5% vs 67.7%	hydrocortisone 50 mg/d for 6h and fludrocortisone days 50*μ*g once daily for 7 days	90-d, 28-d,180-d, ICU discharge and hospital mortality, shock reversal, the time to weaning from mechanical ventilation, mechanical ventilation free days, ICU and hospital LoS, SOFA Score, and adverse events

Venkatesh et al, 2018	Australia and New Zealand	Multicentre double blind randomised placebo controlled trial	3	3658	Septic shock treated with vasopressor and undergoing mechanical ventilation	62.3(14.9) vs 62.7(15.2)	60.4% vs 61.3%	hydrocortisone 200mg /d for 7 days	90-d, 28-d, ICU and hospital mortality, shock reversal, the frequency and duration of mechanical ventilation and RRT, ICU and hospital LoS, receipt of blood transfusion and adverse events

**Table 2:** Different effect-model in each subgroup analysis.

	Studies, n	Patinets, n	Random Effects	Fixed Effects	*P*	*I* ^2^, %	Heterogeneity *P*
Overall	21	9043	0.94(0.89,1.00)	0.93(0.88,0.99)	0.05	2	0.43
Long course of low-dose corticosteroids	18	8665	0.93(0.87,0.98)	0.92(0.86,0.98)	0.01	0	0.52
Short course of high-dose corticosteroid	3	378	1.15(0.94,1.42)	1.17(0.94,1.46)	0.15	0	0.82
before 21s	6	503	0.97(0.74,1.26)	1.01(0.83,1.23)	0.03	0	0.64
after 21s	15	8540	0.93(0.88,0.99)	0.93(0.87,0.99)	0.8	0	0.16
Hydrocortisone concomitant fludrocortisone therapy	2	1540	0.88(0.78,0.99)	0.87(0.78,0.99)	0.03	0	0.79
Hydrocortisone alone therapy	14	7072	0.95(0.89,1.02)	0.94(0.87,1.01)	0.09	0	0.49
Sample size≥400	3	6594	0.90(0.83,0.97)	0.90(0.83,0.98)	<0.001	0	0.67
Sample size<400	18	2448	0.99(0.91,1.08)	0.99(0.90,1.08)	0.77	0	0.46

## Supplementary Materials

Supplementary Materials
* Figure S1:* PRISMA Checklist.* Figure S2:* summary of findings.* Figure S3:* forest plots of comparison corticosteroids versus control of ICU mortality and hospital mortality.* Figure S4:* forest plots of length of hospital stay and ICU stay for all participants.* Figure S5:* forest plots of duration of mechanical ventilation and mechanical ventilation-free days for all participants.* Figure S6:* forest plots of comparison corticosteroids versus control of adverse events.* Figure S7:* forest plots of comparison corticosteroids versus control of 28-day all-cause mortality based on subgroups.
