# Supplementary material for: Reevaluating the Role of Corticosteroids in Septic Shock: An Updated Meta-Analysis of Randomized Controlled Trials
Source: Biomed Res Int. 2019 Jun 10;2019:3175047. doi: 10.1155/2019/3175047 (PMC6590573; doi:10.1155/2019/3175047)

Supplementary Materials

Table S1 Summary of search strategy

Search No	Search strategy*
#1	MeSH descriptor: [steroid] explode all trees
#2	MeSH descriptor: [corticosteroid] explode all trees
#3	steroid* or glucocorticoid* or corticosteroid* or cortisone* or hydrocortisone* or prednisolon* or methylprednisolon* or prednison* or dexamethason* or triamcinolon* in All Text
#4	#1 or #2 or #3
#5	MeSH descriptor: [sepsis] explode all trees
#6	MeSH descriptor: [shock, septic] explode all trees
#7	seps* or septic* in All Text
#8	#5 or #6 or #7
#9	((randomized controlled trial or controlled clinical trial).pt. or randomized.ab. or placebo.ab. or clinical trials as topic.sh. or randomly.ab. or trial.ti.)
#10	#4 and #8 and #9

*This search strategy was adopted for following databases: PubMed, MEDLINE, EMBASE, Web of Science and Cochrane Central Register of Controlled Trials (CENTRAL).

Table S2 Summary of previous research findings

Title	Study (year)	Publish journal	Included trails	Participants	Subgroup analyse	Primary outcomes	Subgroup analyse outcomes	Adverse events	Conclusions
Safety and Efficacy of Corticosteroids for the Treatment of Septic Shock: A Systematic Review and Meta-Analysis	Sligl W, 2009	Clinical Infectious Diseases	8	Adults with septic shock	Responders and non-responders based on corticotropin stimulation test	28-day all-cause mortality (RR, 1.00; 95% CI, 0.84–1.18,P=0.97) Shock reversal (RR, 1.41; 95% CI, 1.22–1.64, P=)	Responders(RR, 0.95; 95% CI, 0.70- 1.28,P=0.72) Nonresponders(RR, 0.90; 95% CI, 0.75–1.07,P=0.23)	Superinfection(RR, 1.11; 95% CI, 0.86–1.42, P=0.42)	Corticosteroid therapy appears to be safe but does not reduce 28-day all-cause mortality rates
CorticosteroidsintheTreatmentof Severe Sepsis and Septic Shock in Adults	Annane, 2009	The Journal of the American Medical Association	22	Severe Sepsis and Septic Shock in Adults	Long course of low-dose and short courses of high-dose corticosteroids	28-day all-cause mortality (RR,0.84; 95% CI, 0.71-1.00; P=.05). Increased 28-day shock reversal(RR, 1.12; 95% CI, 1.02-1.23, P=.02)	Prolonged low-dose corticosteroid suggests benefit on short-term mortality (RR, 0.84; 95%CI, 0.72- 0.97, P=.02)	No increasing the risk of gastroduodenal bleeding, superinfection and neuromuscular weakness. Hyperglycemia (RR, 1.16; 95% CI, 1.07-1.25, P<001) Hypernatremia (RR, 1.61; 95% CI, 1.26-2.06, P<001)	No clear benefit on mortality.
Low-Dose Hydrocortisone Therapy Attenuates Septic Shock in Adult Patients but Does Not Reduce	Wang, 2014	Society of Critical Care Anesthesiologists	8	Septic Shock in Adult	Subgroup analyses for sample size (< 100 or > 100) and quality score (6 or 7)	28-day all-cause mortality (RR,0.84; 95% CI, 0.71-1.00; P=.05). 7-day and 28-day shock reversal (OR = 2.08, 95% CI, 1.58–2.73, P < 0.0001)	No clear benefit on mortality. Benefit on shock reversal	No increasing the risk of gastroduodenal bleeding and superinfection. Hyperglycemia (OR = 2.143, 95% CI, 1.41–3.26, P < 0.0001)	Ameliorates septic shock at 7 and 28 days, but no benefit 28-day mortality

28-Day Mortality: A Meta-Analysis of Randomized Controlled Trials						and (OR = 1.50, 95% CI, 1.12–1.99, P = 0.006).			
Corticosteroids for treating sepsis	Annane, 2015	The Cochrane Collaboration	33	Patients with sepsis	Based on treatment dose/duration, methodological quality and targeted population (sepsis or only septic shock)	28-day all-cause mortality (RR, 0.88; 95% CI 0.78 to 0.99, P=0.01) 7-day and 28-day shock reversal (OR = 1.31, 95% CI 1.14 to 1.51, P = 0.001) and (OR = 1.11, 95% CI 1.02 to 1.21; P = 0.01).	No information only for septic shock.	No information only for septic shock.	Benefit on mortality.
Corticosteroids in septic shock: a systematic review and network meta-analysis	Gibbison, 2017	Critical Care	Complete data from 22 studies and partial data from 1 study.	Septic Shock in Adult	Based on treatment regimen.	No clear evidence that any intervention or treatment regimen is better than any other across the spectrum of mortality. Benefit on shock reversal.	No.	No clear evidence that any one corticosteroid drug or treatment regimen is more likely to be effective reducing the incidence of gastrointestinal bleeding or superinfection in septic shock.	No clear evidence that any one corticosteroid drug or treatment regimen is more likely to be effective in reducing mortality. Hydrocortisone shows more shock reversal.

Fig. S1 PRISMA Checklist.

Section/topic	#	Checklist item	Reported on page #
**TITLE**			
Title	1	Identify the report as a systematic review, meta-analysis, or both.	1
**ABSTRACT**			
Structured summary	2	Provide a structured summary including, as applicable: background; objectives; data sources; study eligibility criteria, participants, and interventions; study appraisal and synthesis methods; results; limitations; conclusions and implications of key findings; systematic review registration number.	3, 4
**INTRODUCTION**			
Rationale	3	Describe the rationale for the review in the context of what is already known.	5
Objectives	4	Provide an explicit statement of questions being addressed with reference to participants, interventions, comparisons, outcomes, and study design (PICOS).	5
**METHODS**			
Protocol and registration	5	Indicate if a review protocol exists, if and where it can be accessed (e.g., Web address), and, if available, provide registration information including registration number.	6
Eligibility criteria	6	Specify study characteristics (e.g., PICOS, length of follow-up) and report characteristics (e.g., years considered, language, publication status) used as criteria for eligibility, giving rationale.	7
Information sources	7	Describe all information sources (e.g., databases with dates of coverage, contact with study authors to identify additional studies) in the search and date last searched.	6
Search	8	Present full electronic search strategy for at least one database, including any limits used, such that it could be	Table S1

		repeated.	
Study selection	9	State the process for selecting studies (i.e., screening, eligibility, included in systematic review, and, if applicable, included in the meta-analysis).	7
Data collection process	10	Describe method of data extraction from reports (e.g., piloted forms, independently, in duplicate) and any processes for obtaining and confirming data from investigators.	7
Data items	11	List and define all variables for which data were sought (e.g., PICOS, funding sources) and any assumptions and simplifications made.	7
Risk of bias in individual studies	12	Describe methods used for assessing risk of bias of individual studies (including specification of whether this was done at the study or outcome level), and how this information is to be used in any data synthesis.	8
Summary measures	13	State the principal summary measures (e.g., risk ratio, difference in means).	8
Synthesis of results	14	Describe the methods of handling data and combining results of studies, if done, including measures of consistency (e.g., I^2) for each meta-analysis.	8
**Section/topic**	**#**	**Checklist item**	**Reported on page #**
Risk of bias across studies	15	Specify any assessment of risk of bias that may affect the cumulative evidence (e.g., publication bias, selective reporting within studies).	8
Additional analyses	16	Describe methods of additional analyses (e.g., sensitivity or subgroup analyses, meta-regression), if done, indicating which were pre-specified.	8-9
**RESULTS**			
Study selection	17	Give numbers of studies screened, assessed for eligibility, and included in the review, with reasons for exclusions at each stage, ideally with a flow diagram.	9 and Fig. 1

Study characteristics	18	For each study, present characteristics for which data were extracted (e.g., study size, PICOS, follow-up period) and provide the citations.	9 and Table 1
Risk of bias within studies	19	Present data on risk of bias of each study and, if available, any outcome level assessment (see item 12).	10 and Fig. 2
Results of individual studies	20	For all outcomes considered (benefits or harms), present, for each study: (a) simple summary data for each intervention group (b) effect estimates and confidence intervals, ideally with a forest plot.	10-12
Synthesis of results	21	Present results of each meta-analysis done, including confidence intervals and measures of consistency.	Table 2
Risk of bias across studies	22	Present results of any assessment of risk of bias across studies (see Item 15).	10
Additional analysis	23	Give results of additional analyses, if done (e.g., sensitivity or subgroup analyses, meta-regression [see Item 16]).	13 and Fig. 3
**DISCUSSION**			
Summary of evidence	24	Summarize the main findings including the strength of evidence for each main outcome; consider their relevance to key groups (e.g., healthcare providers, users, and policy makers).	13-16
Limitations	25	Discuss limitations at study and outcome level (e.g., risk of bias), and at review-level (e.g., incomplete retrieval of identified research, reporting bias).	17
Conclusions	26	Provide a general interpretation of the results in the context of other evidence, and implications for future research.	17
**FUNDING**			
Funding	27	Describe sources of funding for the systematic review and other support (e.g., supply of data); role of funders for the systematic review.	18

From: Moher D, Liberati A, Tetzlaff J, Altman DG, The PRISMA Group (2009). Preferred Reporting Items for Systematic Reviews and Meta-Analyses: The PRISMA Statement. PLoS Med 6(6): e1000097. doi:10.1371/journal.pmed1000097 For more information, visit: **www.prisma-statement.org**.

Fig. S2 Summary of findings table

corticosteroids for septic shock

Patient or population: patients with septic shock
Settings:
Intervention: corticosteroids

Outcomes	Illustrative comparative risks* (95% CI)		Relative effect (95% CI)	No of Participants (studies)	Quality of the evidence (GRADE)	Comments
	Assumed risk	Corresponding risk				
	Control	Corticosteroids				
**28-day all cause mortality**<br>Follow-up: 3 months	**Study population**		**RR 0.93**<br>(0.88 to 0.99)	9043<br>(21 studies)	⊕⊕⊕⊖<br>**moderate**[1]	
	**312 per 1000**	**290 per 1000**<br>(274 to 309)				
	**Moderate**					
	**333 per 1000**	**310 per 1000**<br>(293 to 330)				

*The basis for the **assumed risk** (e.g. the median control group risk across studies) is provided in footnotes. The **corresponding risk** (and its 95% confidence interval) is based on the assumed risk in the comparison group and the **relative effect** of the intervention (and its 95% CI).

CI: Confidence interval; **RR:** Risk ratio;

GRADE Working Group grades of evidence
High quality: Further research is very unlikely to change our confidence in the estimate of effect.
Moderate quality: Further research is likely to have an important impact on our confidence in the estimate of effect and may change the estimate.
Low quality: Further research is very likely to have an important impact on our confidence in the estimate of effect and is likely to change the estimate.
Very low quality: We are very uncertain about the estimate.

[1] Benefit-to-riskratio of some studies, remains controversia.

Fig. S3 Forest plots of comparison corticosteroids versus control of ICU mortality(a) and hospital mortality(b).

a.

Study or Subgroup	Corticosteroids Events	Corticosteroids Total	Control Events	Control Total	Weight	Risk Ratio M-H, Fixed, 95% CI	Year
Bollaert 1998	8	22	12	19	4.2%	0.58 [0.30, 1.10]	1998
Briegel1999	4	20	6	20	1.9%	0.67 [0.22, 2.01]	1999
Chawla 1999	6	23	8	21	2.7%	0.68 [0.28, 1.65]	1999
Annane 2002	90	150	101	149	32.7%	0.89 [0.75, 1.05]	2002
Sprung 2008	102	251	89	248	28.9%	1.13 [0.91, 1.42]	2008
Hu 2009	4	38	6	39	1.9%	0.68 [0.21, 2.23]	2009
Arabi 2010	24	39	24	36	8.1%	0.92 [0.66, 1.30]	2010
Gordon 2014	7	31	8	30	2.6%	0.85 [0.35, 2.04]	2014
Gordon2 2016	24	101	27	103	8.6%	0.91 [0.56, 1.46]	2016
Gordon 2016	32	101	26	104	8.3%	1.27 [0.82, 1.97]	2016
**Total (95% CI)**		**776**		**769**	**100.0%**	**0.97 [0.86, 1.09]**	
Total events	301		307				

Heterogeneity: Chi² = 8.41, df = 9 (P = 0.49); I² = 0%

Test for overall effect: Z = 0.58 (P = 0.56)

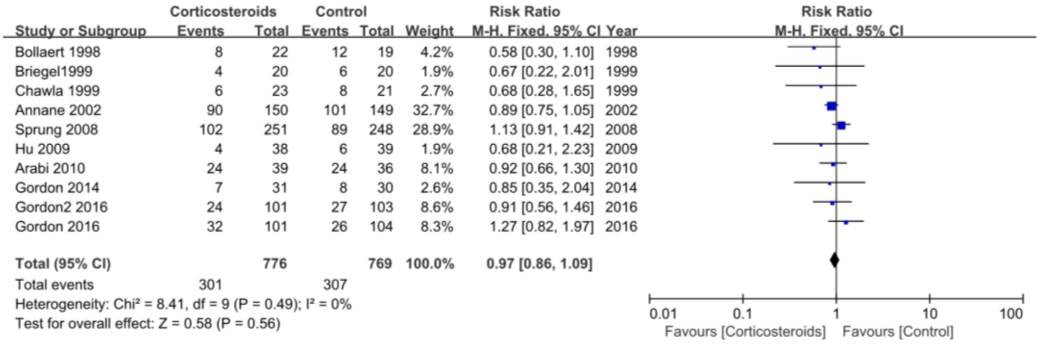

b.

Study or Subgroup	Corticosteroids Events	Corticosteroids Total	Control Events	Control Total	Weight	Risk Ratio M-H, Fixed, 95% CI	Year
Sprung 1984	33	43	11	16	3.4%	1.12 [0.77, 1.61]	1984
Luce 1988	22	38	20	37	4.3%	1.07 [0.72, 1.60]	1988
Bollaert 1998	8	22	12	19	2.8%	0.58 [0.30, 1.10]	1998
Chawla 1999	6	23	10	21	2.2%	0.55 [0.24, 1.25]	1999
Briegel1999	5	20	6	20	1.3%	0.83 [0.30, 2.29]	1999
Annane 2002	95	150	103	149	22.1%	0.92 [0.78, 1.08]	2002
Sprung 2008	111	251	100	248	21.6%	1.10 [0.89, 1.35]	2008
Arabi 2010	34	39	32	36	7.1%	0.98 [0.83, 1.16]	2010
Gordon 2014	8	31	9	30	2.0%	0.86 [0.38, 1.93]	2014
Pόvoa2 2015	31	403	29	442	5.9%	1.17 [0.72, 1.91]	2015
Pόvoa 2015	35	436	33	414	7.3%	1.01 [0.64, 1.59]	2015
Gordon 2016	35	101	33	104	7.0%	1.09 [0.74, 1.61]	2016
Gordon2 2016	31	101	29	103	6.2%	1.09 [0.71, 1.67]	2016
Tongyoo 2016	11	78	13	76	2.8%	0.82 [0.39, 1.72]	2016
Lv 2017	23	58	19	60	4.0%	1.25 [0.77, 2.04]	2017
**Total (95% CI)**		**1794**		**1775**	**100.0%**	**1.01 [0.92, 1.11]**	
Total events	488		459				

Heterogeneity: Chi² = 9.43, df = 14 (P = 0.80); I² = 0%

Test for overall effect: Z = 0.19 (P = 0.85)

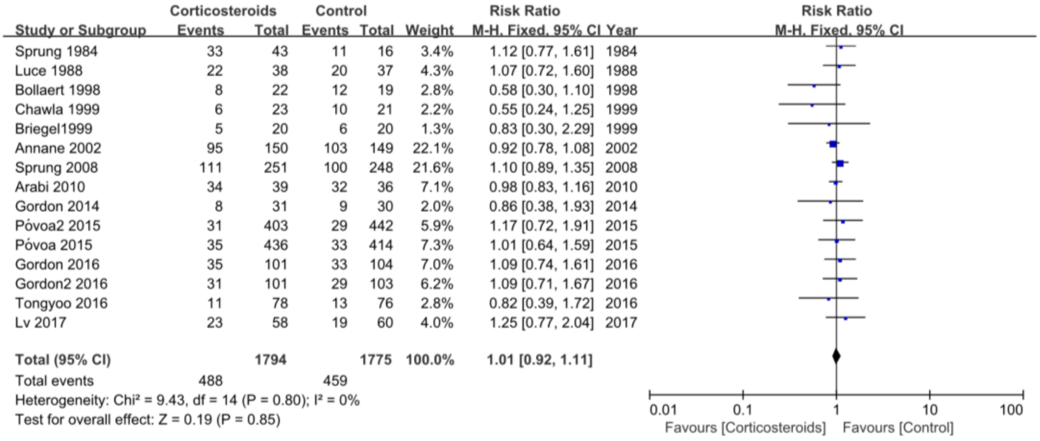

Fig. S4 Forest plots of comparison corticosteroids versus control of length of ICU stay (a) and hospital stay for all participants (b).

a.

	Corticosteroids			Control				Mean Difference	
Study or Subgroup	Mean	SD	Total	Mean	SD	Total	Weight	IV, Fixed, 95% CI	Year
Bollaert 1998	26	24	22	19	18	19	0.3%	7.00 [-5.89, 19.89]	1998
Briegel1999	29	16	20	38	24	20	0.3%	-9.00 [-21.64, 3.64]	1999
Chawla 1999	7.4	6.2	23	10.7	7.6	21	2.7%	-3.30 [-7.42, 0.82]	1999
Annane 2002	22	24	150	25.5	18	149	2.0%	-3.50 [-8.31, 1.31]	2002
Sprung 2008	19	31	251	18	17	248	2.4%	1.00 [-3.38, 5.38]	2008
Hu 2009	4.18	2.86	38	5.36	2.48	39	32.5%	-1.18 [-2.38, 0.02]	2009
Arabi 2010	10.5	6.7	39	9.7	6.5	36	5.2%	0.80 [-2.19, 3.79]	2010
Gordon 2014	14.3	14.6	31	19.5	14.9	30	0.8%	-5.20 [-12.61, 2.21]	2014
Gordon2 2016	5	8.9	101	6	5.93	103	10.8%	-1.00 [-3.08, 1.08]	2016
Gordon 2016	6	5.2	101	7	8.15	104	13.4%	-1.00 [-2.87, 0.87]	2016
Lv 2017	10.9	17.5	58	10.2	13.1	60	1.5%	0.70 [-4.89, 6.29]	2017
Annane 2018	19	25	614	17	21	627	7.0%	2.00 [-0.57, 4.57]	2018
Venkatesh 2018	10	18.53	1832	12	26.69	1826	21.0%	-2.00 [-3.49, -0.51]	2018
**Total (95% CI)**			**3280**			**3282**	**100.0%**	**-1.04 [-1.72, -0.36]**	

Heterogeneity: Chi² = 16.07, df = 12 (P = 0.19); I² = 25%
Test for overall effect: Z = 2.99 (P = 0.003)

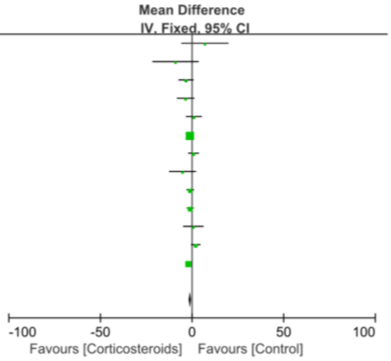

b.

	Corticosteroids			Control				Mean Difference	
Study or Subgroup	Mean	SD	Total	Mean	SD	Total	Weight	IV, Fixed, 95% CI	Year
Briegel1999	16.9	13.3	20	21	14.5	20	8.2%	-4.10 [-12.72, 4.52]	1999
Annane 2002	20	21	150	25	22	149	25.7%	-5.00 [-9.88, -0.12]	2002
Sprung 2008	34	41	251	34	37	248	13.0%	0.00 [-6.85, 6.85]	2008
Arabi 2010	22	13.4	39	26.4	23.6	36	7.9%	-4.40 [-13.18, 4.38]	2010
Gordon 2014	34	32.8	31	35.9	25	30	2.9%	-1.90 [-16.51, 12.71]	2014
Gordon 2016	13	17.79	101	17	22.98	104	19.3%	-4.00 [-9.62, 1.62]	2016
Gordon2 2016	16	25.2	101	15	20.76	103	15.2%	1.00 [-5.34, 7.34]	2016
Lv 2017	23.7	26.8	58	21.7	21.7	60	7.9%	2.00 [-6.82, 10.82]	2017
**Total (95% CI)**			**751**			**750**	**100.0%**	**-2.49 [-4.96, -0.02]**	

Heterogeneity: Chi² = 4.28, df = 7 (P = 0.75); I² = 0%
Test for overall effect: Z = 1.97 (P = 0.05)

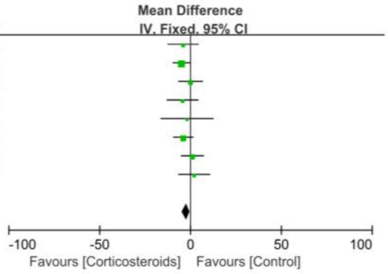

Fig. S5 Forest plots of comparison corticosteroids versus control of mechanical ventilation free days (**a**) and duration of mechanical ventilation (**b**) for all participants.

a.

	Corticosteroids			Control				Mean Difference	
Study or Subgroup	Mean	SD	Total	Mean	SD	Total	Weight	IV, Fixed, 95% CI	Year
Arabi 2010	6.7	7.7	39	8.1	10.9	36	5.5%	-1.40 [-5.70, 2.90]	2010
Tongyoo 2016	11	11	78	10	11	76	8.4%	1.00 [-2.47, 4.47]	2016
Venkatesh 2018	61.2	35.6	1832	59.1	36.1	1826	18.7%	2.10 [-0.22, 4.42]	2018
Annane 2018	11	11	614	10	11	627	67.5%	1.00 [-0.22, 2.22]	2018
**Total (95% CI)**			**2563**			**2565**	**100.0%**	**1.07 [0.07, 2.08]**	
Heterogeneity: Chi² = 2.03, df = 3 (P = 0.57); I² = 0%									
Test for overall effect: Z = 2.10 (P = 0.04)									

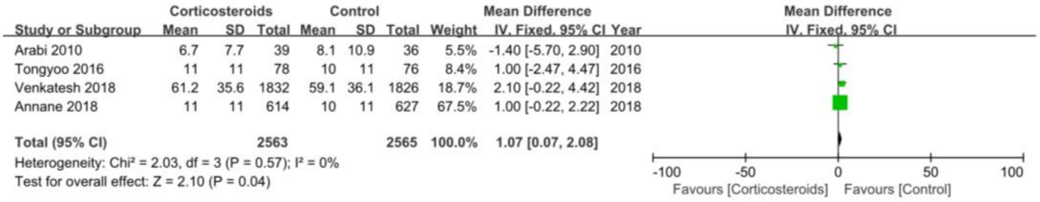

b.

	Corticosteroids			Control				Mean Difference	
Study or Subgroup	Mean	SD	Total	Mean	SD	Total	Weight	IV, Fixed, 95% CI	Year
Briegel1999	18	0	20	38	0	20		Not estimable	1999
Cicarelli 2007	3.4	2.5	14	4	3.2	15	11.6%	-0.60 [-2.68, 1.48]	2007
Gordon 2016	5	5.93	101	6	6.67	104	16.9%	-1.00 [-2.73, 0.73]	2016
Gordon2 2016	5	10.38	101	5	10.38	103	6.2%	0.00 [-2.85, 2.85]	2016
Venkatesh 2018	6	11.13	1832	7	15.57	1826	65.3%	-1.00 [-1.88, -0.12]	2018
**Total (95% CI)**			**2048**			**2048**	**100.0%**	**-0.89 [-1.60, -0.18]**	
Heterogeneity: Chi² = 0.53, df = 3 (P = 0.91); I² = 0%									
Test for overall effect: Z = 2.46 (P = 0.01)									

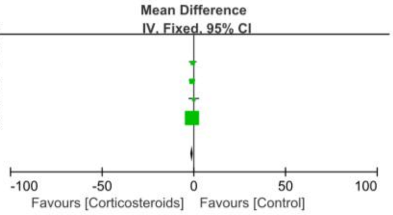

Fig. S6 Forest plots of comparison corticosteroids versus control of adverse events, including gastroduodenal bleeding (**a**), superinfections (**b**) and hyperglycaemia(**c**).

a.

Study or Subgroup	Corticosteroids Events	Corticosteroids Total	Control Events	Control Total	Weight	Risk Ratio M-H, Fixed, 95% CI	Year
Sprung 1984	1	43	2	16	3.0%	0.19 [0.02, 1.91]	1984
Luce 1988	18	38	16	37	16.9%	1.10 [0.67, 1.80]	1988
Bollaert 1998	1	22	3	19	3.4%	0.29 [0.03, 2.54]	1998
Briegel1999	1	20	0	20	0.5%	3.00 [0.13, 69.52]	1999
Chawla 1999	1	23	2	21	2.2%	0.46 [0.04, 4.68]	1999
Annane 2002	11	150	8	149	8.4%	1.37 [0.57, 3.30]	2002
Sprung 2008	15	251	13	248	13.7%	1.14 [0.55, 2.35]	2008
Arabi 2010	13	39	4	36	4.3%	3.00 [1.08, 8.36]	2010
Venkatesh 2018	2	1832	1	1826	1.0%	1.99 [0.18, 21.96]	2018
Annane 2018	39	614	45	627	46.5%	0.89 [0.58, 1.34]	2018
**Total (95% CI)**		**3032**		**2999**	**100.0%**	**1.06 [0.82, 1.37]**	
Total events	102		94				

Heterogeneity: Chi² = 9.77, df = 9 (P = 0.37); I² = 8%
Test for overall effect: Z = 0.44 (P = 0.66)

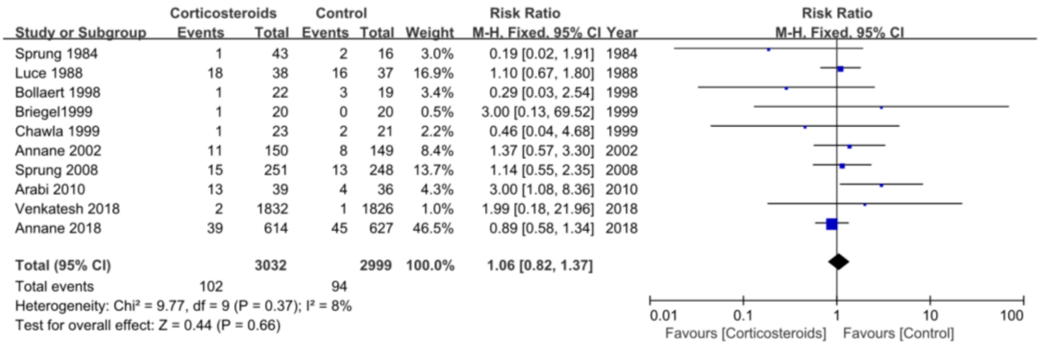

b.

Study or Subgroup	Corticosteroids Events	Corticosteroids Total	Control Events	Control Total	Weight	Risk Ratio M-H, Fixed, 95% CI	Year
Sprung 1984	11	43	1	16	0.2%	4.09 [0.57, 29.20]	1984
Bone 1987	29	130	30	114	5.3%	0.85 [0.54, 1.32]	1987
Luce 1988	3	38	4	37	0.7%	0.73 [0.18, 3.04]	1988
Bollaert 1998	7	22	9	19	1.6%	0.67 [0.31, 1.46]	1998
Briegel1999	10	20	7	20	1.2%	1.43 [0.68, 3.00]	1999
Chawla 1999	4	23	5	21	0.9%	0.73 [0.23, 2.36]	1999
Annane 2002	22	150	27	149	4.5%	0.81 [0.48, 1.35]	2002
Cicarelli 2007	0	14	1	15	0.2%	0.36 [0.02, 8.07]	2007
Sprung 2008	78	251	61	248	10.1%	1.26 [0.95, 1.68]	2008
Arabi 2010	22	39	18	36	3.1%	1.13 [0.74, 1.73]	2010
Annane 2018	191	614	178	627	29.0%	1.10 [0.92, 1.30]	2018
Venkatesh 2018	262	1832	262	1826	43.3%	1.00 [0.85, 1.17]	2018
**Total (95% CI)**		**3176**		**3128**	**100.0%**	**1.04 [0.94, 1.15]**	
Total events	639		603				

Heterogeneity: Chi² = 9.11, df = 11 (P = 0.61); I² = 0%
Test for overall effect: Z = 0.82 (P = 0.41)

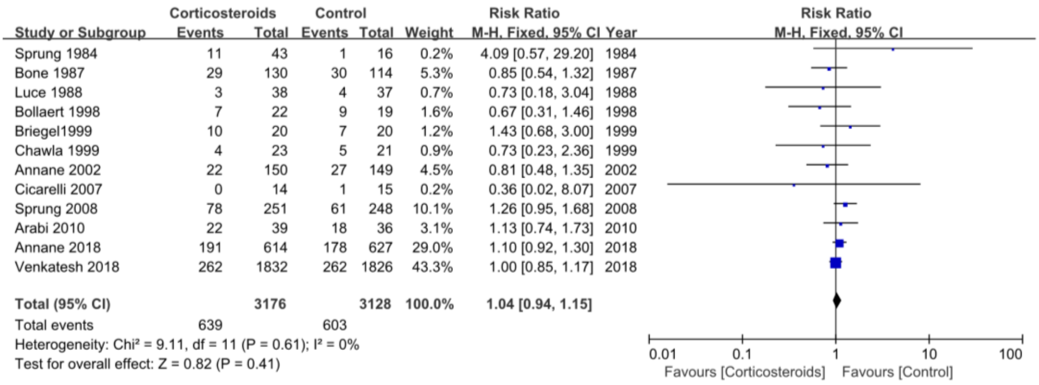

c.

Study or Subgroup	Corticosteroids Events	Corticosteroids Total	Control Events	Control Total	Weight	Risk Ratio M-H, Fixed, 95% CI	Year
Sprung 1984	4	43	0	16	0.1%	3.48 [0.20, 61.18]	1984
Luce 1988	16	38	15	37	1.8%	1.04 [0.61, 1.78]	1988
Bollaert 1998	3	22	3	19	0.4%	0.86 [0.20, 3.79]	1998
Annane 2002	130	150	111	149	13.3%	1.16 [1.04, 1.30]	2002
Sprung 2008	186	251	161	248	19.4%	1.14 [1.02, 1.28]	2008
Arabi 2010	34	39	25	36	3.1%	1.26 [0.98, 1.61]	2010
Annane 2018	547	614	520	627	61.5%	1.07 [1.03, 1.12]	2018
Venkatesh 2018	6	1832	3	1826	0.4%	1.99 [0.50, 7.96]	2018
**Total (95% CI)**		**2989**		**2958**	**100.0%**	**1.11 [1.06, 1.16]**	
Total events	926		838				

Heterogeneity: Chi² = 5.26, df = 7 (P = 0.63); I² = 0%
Test for overall effect: Z = 4.82 (P < 0.00001)

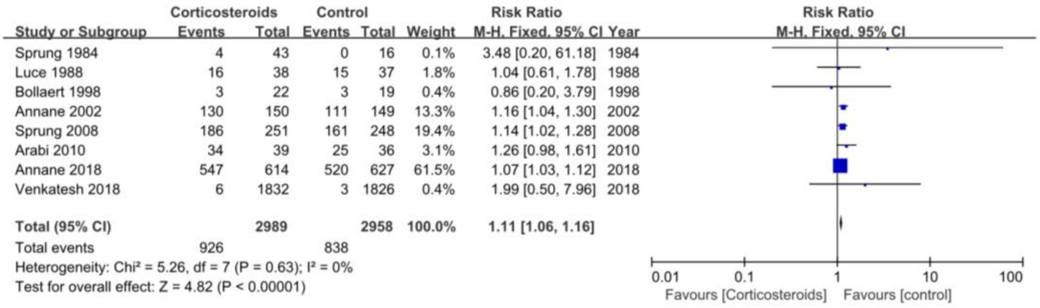

Fig. S7 Forest plots of comparison corticosteroids versus control of 28-day all-cause mortality by subgroups based on treatment dose and course (**a**), whether concomitant mineralocorticoid (**b**), date of publication (**c**), and size sample(**d**).

a.

Study or Subgroup	Corticosteroids Events	Corticosteroids Total	Control Events	Control Total	Weight	Risk Ratio M-H, Random, 95% CI	Year
**8.1.1 Long course of low-dose corticosteroids**							
Bollaert 1998	7	22	12	19	0.7%	0.50 [0.25, 1.02]	1998
Briegel1999	3	20	4	20	0.2%	0.75 [0.19, 2.93]	1999
Chawla 1999	6	23	10	21	0.5%	0.55 [0.24, 1.25]	1999
Annane 2002	82	150	91	149	9.2%	0.90 [0.74, 1.09]	2002
Oppert 2005	7	18	11	23	0.7%	0.81 [0.40, 1.67]	2005
Mussack 2005	3	12	5	12	0.3%	0.60 [0.18, 1.97]	2005
Cicarelli 2007	7	14	12	15	1.1%	0.63 [0.35, 1.12]	2007
Sprung 2008	86	251	78	248	5.6%	1.09 [0.85, 1.40]	2008
Hu 2009	4	38	6	39	0.3%	0.68 [0.21, 2.23]	2009
Arabi 2010	33	39	26	36	6.0%	1.17 [0.92, 1.49]	2010
Gordon 2014	7	31	7	30	0.4%	0.97 [0.39, 2.43]	2014
Póvoa2 2015	95	403	103	442	5.9%	1.01 [0.79, 1.29]	2015
Póvoa 2015	108	436	122	414	7.1%	0.84 [0.67, 1.05]	2015
Tongyoo 2016	19	78	24	76	1.4%	0.77 [0.46, 1.29]	2016
Gordon 2016	33	101	30	104	2.1%	1.13 [0.75, 1.71]	2016
Gordon2 2016	29	101	27	103	1.8%	1.10 [0.70, 1.71]	2016
Lv 2017	23	58	19	60	1.5%	1.25 [0.77, 2.04]	2017
Venkatesh 2018	410	1832	448	1826	23.4%	0.91 [0.81, 1.03]	2018
Annane 2018	207	614	244	627	15.3%	0.87 [0.75, 1.00]	2018
Doluee 2018	54	80	58	80	8.4%	0.93 [0.76, 1.14]	2018
**Subtotal (95% CI)**		**4321**		**4344**	**91.9%**	**0.93 [0.87, 0.98]**	
Total events	1223		1337				
Heterogeneity: Tau² = 0.00; Chi² = 18.10, df = 19 (P = 0.52); I² = 0%							
Test for overall effect: Z = 2.50 (P = 0.01)							
**8.1.2 Short course of high-dose corticosteroid**							
Sprung 1984	33	43	11	16	2.6%	1.12 [0.77, 1.61]	1984
Bone 1987	54	130	38	114	3.3%	1.25 [0.90, 1.73]	1987
Luce 1988	22	38	20	37	2.2%	1.07 [0.72, 1.60]	1988
**Subtotal (95% CI)**		**211**		**167**	**8.1%**	**1.15 [0.94, 1.42]**	
Total events	109		69				
Heterogeneity: Tau² = 0.00; Chi² = 0.39, df = 2 (P = 0.82); I² = 0%							
Test for overall effect: Z = 1.34 (P = 0.18)							
**Total (95% CI)**		**4532**		**4511**	**100.0%**	**0.94 [0.89, 1.00]**	
Total events	1332		1406				
Heterogeneity: Tau² = 0.00; Chi² = 22.44, df = 22 (P = 0.43); I² = 2%							
Test for overall effect: Z = 1.93 (P = 0.05)							
Test for subgroup differences: Chi² = 3.93, df = 1 (P = 0.05), I² = 74.5%							

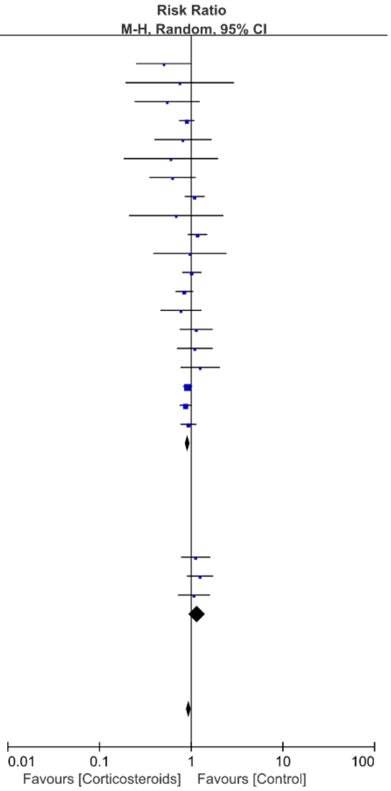

b.

Study or Subgroup	Corticosteroids Events	Corticosteroids Total	Control Events	Control Total	Weight	Risk Ratio M-H, Fixed, 95% CI	Year
**8.3.1 Hydrocortisone concomitant fludrocortisone therapy**							
Annane 2002	82	150	91	149	6.9%	0.90 [0.74, 1.09]	2002
Annane 2018	207	614	244	627	18.3%	0.87 [0.75, 1.00]	2018
**Subtotal (95% CI)**		**764**		**776**	**25.3%**	**0.87 [0.78, 0.99]**	
Total events	289		335				
Heterogeneity: Chi² = 0.07, df = 1 (P = 0.79); I² = 0%							
Test for overall effect: Z = 2.20 (P = 0.03)							
**8.3.2 Hydrocortisone therapy**							
Bollaert 1998	7	22	12	19	1.0%	0.50 [0.25, 1.02]	1998
Briegel1999	3	20	4	20	0.3%	0.75 [0.19, 2.93]	1999
Chawla 1999	6	23	10	21	0.8%	0.55 [0.24, 1.25]	1999
Oppert 2005	7	18	11	23	0.7%	0.81 [0.40, 1.67]	2005
Sprung 2008	86	251	78	248	6.0%	1.09 [0.85, 1.40]	2008
Hu 2009	4	38	6	39	0.4%	0.68 [0.21, 2.23]	2009
Arabi 2010	33	39	26	36	2.1%	1.17 [0.92, 1.49]	2010
Gordon 2014	7	31	7	30	0.5%	0.97 [0.39, 2.43]	2014
Póvoa 2015	108	436	122	414	9.5%	0.84 [0.67, 1.05]	2015
Póvoa2 2015	95	403	103	442	7.5%	1.01 [0.79, 1.29]	2015
Tongyoo 2016	19	78	24	76	1.8%	0.77 [0.46, 1.29]	2016
Gordon 2016	33	101	30	104	2.2%	1.13 [0.75, 1.71]	2016
Gordon2 2016	29	101	27	103	2.0%	1.10 [0.70, 1.71]	2016
Lv 2017	23	58	19	60	1.4%	1.25 [0.77, 2.04]	2017
Venkatesh 2018	410	1832	448	1826	34.1%	0.91 [0.81, 1.03]	2018
Doluee 2018	54	80	58	80	4.4%	0.93 [0.76, 1.14]	2018
**Subtotal (95% CI)**		**3531**		**3541**	**74.7%**	**0.94 [0.87, 1.01]**	
Total events	924		985				
Heterogeneity: Chi² = 14.48, df = 15 (P = 0.49); I² = 0%							
Test for overall effect: Z = 1.67 (P = 0.09)							
**Total (95% CI)**		**4295**		**4317**	**100.0%**	**0.92 [0.87, 0.98]**	
Total events	1213		1320				
Heterogeneity: Chi² = 15.83, df = 17 (P = 0.54); I² = 0%							
Test for overall effect: Z = 2.50 (P = 0.01)							
Test for subgroup differences: Chi² = 0.96, df = 1 (P = 0.33), I² = 0%							

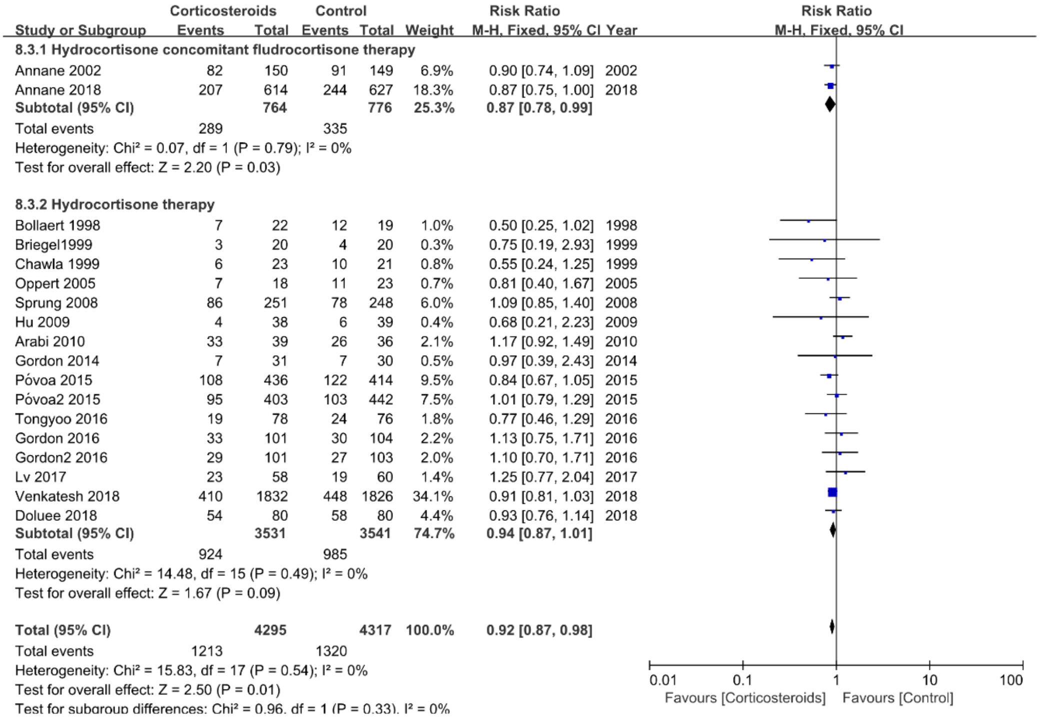

c.

Study or Subgroup	Corticosteroids Events	Corticosteroids Total	Control Events	Control Total	Weight	Risk Ratio M-H, Random, 95% CI	Year
**8.2.1 after 21s**							
Annane 2002	82	150	91	149	9.2%	0.90 [0.74, 1.09]	2002
Mussack 2005	3	12	5	12	0.3%	0.60 [0.18, 1.97]	2005
Oppert 2005	7	18	11	23	0.7%	0.81 [0.40, 1.67]	2005
Cicarelli 2007	7	14	12	15	1.1%	0.63 [0.35, 1.12]	2007
Sprung 2008	86	251	78	248	5.6%	1.09 [0.85, 1.40]	2008
Hu 2009	4	38	6	39	0.3%	0.68 [0.21, 2.23]	2009
Arabi 2010	33	39	26	36	6.0%	1.17 [0.92, 1.49]	2010
Gordon 2014	7	31	7	30	0.4%	0.97 [0.39, 2.43]	2014
Póvoa2 2015	95	403	103	442	5.9%	1.01 [0.79, 1.29]	2015
Póvoa 2015	108	436	122	414	7.1%	0.84 [0.67, 1.05]	2015
Gordon 2016	33	101	30	104	2.1%	1.13 [0.75, 1.71]	2016
Tongyoo 2016	19	78	24	76	1.4%	0.77 [0.46, 1.29]	2016
Gordon2 2016	29	101	27	103	1.8%	1.10 [0.70, 1.71]	2016
Lv 2017	23	58	19	60	1.5%	1.25 [0.77, 2.04]	2017
Doluee 2018	54	80	58	80	8.4%	0.93 [0.76, 1.14]	2018
Venkatesh 2018	410	1832	448	1826	23.4%	0.91 [0.81, 1.03]	2018
Annane 2018	207	614	244	627	15.3%	0.87 [0.75, 1.00]	2018
**Subtotal (95% CI)**		**4256**		**4284**	**90.4%**	**0.93 [0.88, 0.99]**	
Total events	1207		1311				
Heterogeneity: Tau² = 0.00; Chi² = 13.49, df = 16 (P = 0.64); I² = 0%							
Test for overall effect: Z = 2.23 (P = 0.03)							
**8.2.2 before 21s**							
Sprung 1984	33	43	11	16	2.6%	1.12 [0.77, 1.61]	1984
Bone 1987	54	130	38	114	3.3%	1.25 [0.90, 1.73]	1987
Luce 1988	22	38	20	37	2.2%	1.07 [0.72, 1.60]	1988
Bollaert 1998	7	22	12	19	0.7%	0.50 [0.25, 1.02]	1998
Briegel1999	3	20	4	20	0.2%	0.75 [0.19, 2.93]	1999
Chawla 1999	6	23	10	21	0.5%	0.55 [0.24, 1.25]	1999
**Subtotal (95% CI)**		**276**		**227**	**9.6%**	**0.97 [0.74, 1.26]**	
Total events	125		95				
Heterogeneity: Tau² = 0.04; Chi² = 8.01, df = 5 (P = 0.16); I² = 38%							
Test for overall effect: Z = 0.26 (P = 0.80)							
**Total (95% CI)**		**4532**		**4511**	**100.0%**	**0.94 [0.89, 1.00]**	
Total events	1332		1406				
Heterogeneity: Tau² = 0.00; Chi² = 22.44, df = 22 (P = 0.43); I² = 2%							
Test for overall effect: Z = 1.93 (P = 0.05)							
Test for subgroup differences: Chi² = 0.06, df = 1 (P = 0.81), I² = 0%							

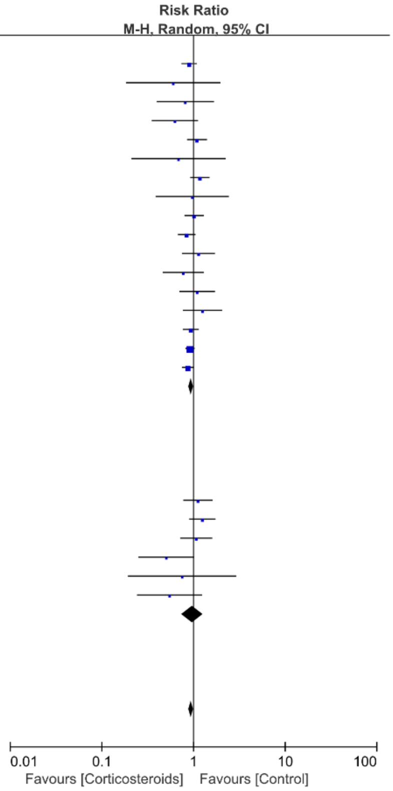

d.

Study or Subgroup	Corticosteroids Events	Corticosteroids Total	Control Events	Control Total	Weight	Risk Ratio M-H, Fixed, 95% CI	Year
**8.4.1 Sample size>400**							
Póvoa2 2015	108	436	122	414	8.9%	0.84 [0.67, 1.05]	2015
Póvoa 2015	95	403	103	442	7.0%	1.01 [0.79, 1.29]	2015
Annane 2018	207	614	244	627	17.1%	0.87 [0.75, 1.00]	2018
Venkatesh 2018	410	1832	448	1826	31.9%	0.91 [0.81, 1.03]	2018
**Subtotal (95% CI)**		**3285**		**3309**	**64.9%**	**0.90 [0.83, 0.98]**	
Total events	820		917				
Heterogeneity: Chi² = 1.56, df = 3 (P = 0.67); I² = 0%							
Test for overall effect: Z = 2.54 (P = 0.01)							
**8.4.3 Sample size<400**							
Sprung 1984	33	43	11	16	1.1%	1.12 [0.77, 1.61]	1984
Bone 1987	54	130	38	114	2.9%	1.25 [0.90, 1.73]	1987
Luce 1988	22	38	20	37	1.4%	1.07 [0.72, 1.60]	1988
Bollaert 1998	7	22	12	19	0.9%	0.50 [0.25, 1.02]	1998
Briegel1999	3	20	4	20	0.3%	0.75 [0.19, 2.93]	1999
Chawla 1999	6	23	10	21	0.7%	0.55 [0.24, 1.25]	1999
Annane 2002	82	150	91	149	6.5%	0.90 [0.74, 1.09]	2002
Mussack 2005	3	12	5	12	0.4%	0.60 [0.18, 1.97]	2005
Oppert 2005	7	18	11	23	0.7%	0.81 [0.40, 1.67]	2005
Cicarelli 2007	7	14	12	15	0.8%	0.63 [0.35, 1.12]	2007
Sprung 2008	86	251	78	248	5.6%	1.09 [0.85, 1.40]	2008
Hu 2009	4	38	6	39	0.4%	0.68 [0.21, 2.23]	2009
Arabi 2010	33	39	26	36	1.9%	1.17 [0.92, 1.49]	2010
Gordon 2014	7	31	7	30	0.5%	0.97 [0.39, 2.43]	2014
Gordon 2016	29	101	27	103	1.9%	1.10 [0.70, 1.71]	2016
Gordon2 2016	29	101	27	103	1.9%	1.10 [0.70, 1.71]	2016
Tongyoo 2016	19	78	24	76	1.7%	0.77 [0.46, 1.29]	2016
Lv 2017	23	58	19	60	1.3%	1.25 [0.77, 2.04]	2017
Doluee 2018	54	80	58	80	4.1%	0.93 [0.76, 1.14]	2018
**Subtotal (95% CI)**		**1247**		**1201**	**35.1%**	**0.99 [0.90, 1.08]**	
Total events	508		486				
Heterogeneity: Chi² = 17.88, df = 18 (P = 0.46); I² = 0%							
Test for overall effect: Z = 0.30 (P = 0.77)							
**Total (95% CI)**		**4532**		**4510**	**100.0%**	**0.93 [0.88, 0.99]**	
Total events	1328		1403				
Heterogeneity: Chi² = 22.11, df = 22 (P = 0.45); I² = 0%							
Test for overall effect: Z = 2.30 (P = 0.02)							
Test for subgroup differences: Chi² = 2.12, df = 1 (P = 0.15), I² = 52.9%							

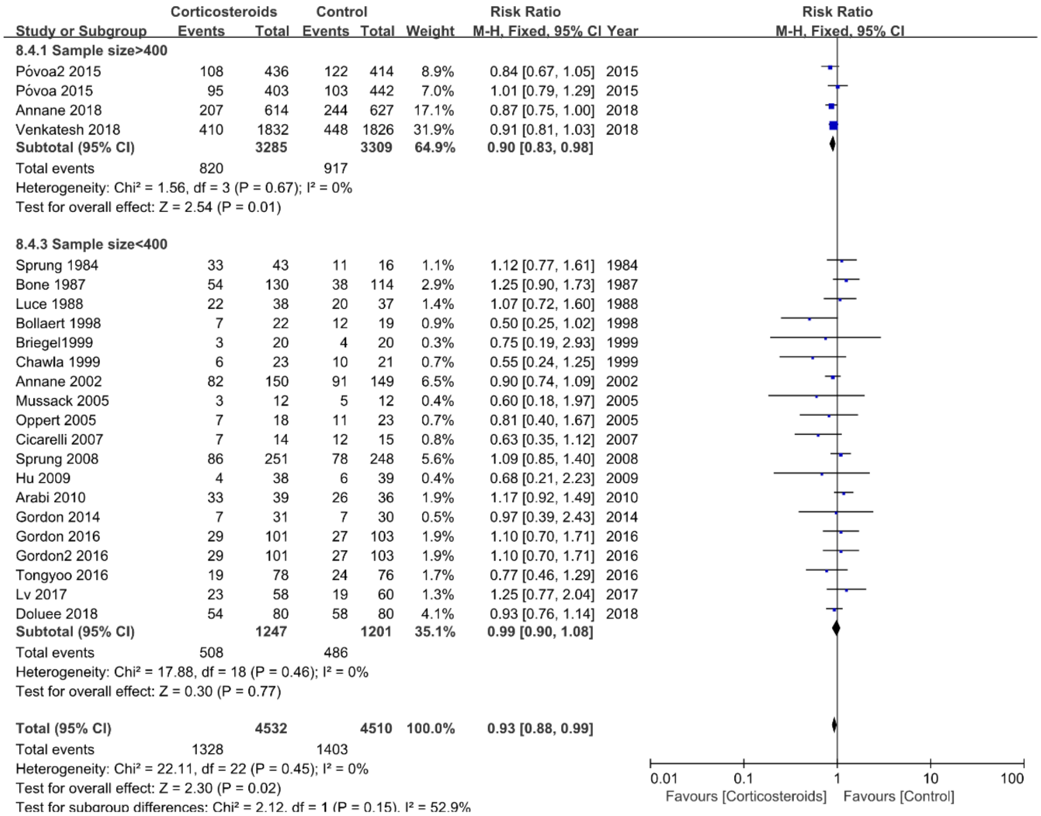

Supplement: Supplementary Materials — Figure S1: PRISMA Checklist. Figure S2: summary of findings. Figure S3: forest plots of comparison corticosteroids versus control of ICU mortality and hospital mortality. Figure S4: forest plots of length of hospital stay and ICU stay for all participants. Figure S5: forest plots of duration of mechanical ventilation and mechanical ventilation-free days for all participants. Figure S6: forest plots of comparison corticosteroids versus control of adverse events. Figure S7: forest plots of comparison corticosteroids versus control of 28-day all-cause mortality based on subgroups. [file 3175047.f1.pdf]
